# Behavioral Abnormality Induced by Enhanced Hypothalamo-Pituitary-Adrenocortical Axis Activity under Dietary Zinc Deficiency and Its Usefulness as a Model

**DOI:** 10.3390/ijms17071149

**Published:** 2016-07-16

**Authors:** Atsushi Takeda, Haruna Tamano, Ryusuke Nishio, Taku Murakami

**Affiliations:** Department of Neurophysiology, School of Pharmaceutical Sciences, University of Shizuoka, 52-1 Yada, Suruga-ku, Shizuoka 422-8526, Japan; tamano@u-shizuoka-ken.ac.jp (H.T.); s16114@u-shizuoka-ken.ac.jp (R.N.); s16115@u-shizuoka-ken.ac.jp (T.M.)

**Keywords:** zinc deficiency, glucocorticoid, hippocampus, glutamate excitotoxicity, behavioral and psychological symptoms of dementia, herbal medicine

## Abstract

Dietary zinc deficiency increases glucocorticoid secretion from the adrenal cortex via enhanced hypothalamo-pituitary-adrenocortical (HPA) axis activity and induces neuropsychological symptoms, i.e., behavioral abnormality. Behavioral abnormality is due to the increase in glucocorticoid secretion rather than disturbance of brain zinc homeostasis, which occurs after the increase in glucocorticoid secretion. A major target of glucocorticoids is the hippocampus and their actions are often associated with disturbance of glutamatergic neurotransmission, which may be linked to behavioral abnormality, such as depressive symptoms and aggressive behavior under zinc deficiency. Glucocorticoid-mediated disturbance of glutamatergic neurotransmission in the hippocampus is also involved in the pathophysiology of, not only psychiatric disorders, such as depression, but also neurodegenerative disorders, e.g., Alzheimer’s disease. The evidence suggests that zinc-deficient animals are models for behavioral and psychological symptoms of dementia (BPSD), as well as depression. To understand validity to apply zinc-deficient animals as a behavioral abnormality model, this paper deals with the effect of antidepressive drugs and herbal medicines on hippocampal dysfunctions and behavioral abnormality, which are induced by enhanced HPA axis activity under dietary zinc deficiency.

## 1. Introduction

**Figure 1:**
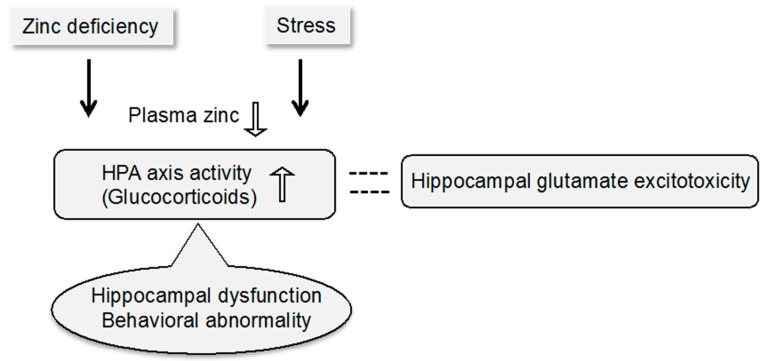
Behavioral abnormality, which is induced by enhanced HPA axis activity under dietary zinc deficiency, as models for depressive symptoms and BPSD. The black arrow, causative action; the white arrow, decreased and increased concentrations; dashed line, close relationship.

Brain zinc homeostasis is critical for brain function [1]. However, the hormones for regulating zinc homeostasis are unknown and the mechanism for regulating zinc homeostasis remains to be clarified. Dietary zinc deficiency leads to a reduction in total food intake. The reduction occurs within approximately three days [2]. The reduction in plasma zinc level also occurs at this time (Figure 1) [3]. Although zinc deficiency-induced anorexia is well known [4], the mechanism is unclear. Zinc deficiency elevates the hypothalamo-pituitary-adrenocortical (HPA) axis activity, followed by an increase in glucocorticoid secretion from the adrenal cortex [5,6], which is involved in stress response (Figure 1) [7]. The continuous increase in plasma corticosterone concentration is observed after daily administration of a zinc-deficient diet [8,9]. Brain zinc homeostasis is resistant to dietary zinc deficiency. However, chronic zinc deficiency decreases extracellular zinc concentration in the hippocampus and then decreases zinc concentration in the synaptic vesicles [10,11]. Free Zn^2+^ may be the most responsive to zinc deficiency, and the decrease in plasma Zn^2+^ might be linked to corticosterone secretion.

All neurons have glutamate receptors in the brain. The extracellular concentration of glutamate is approximately 2 μM in the brain, while glutamate concentration in the synaptic vesicles of glutamatergic neurons reaches approximately 100 mM [12]. Extracellular glutamate signaling is critical for not only synaptic function such as synaptic plasticity but also synaptic dysfunction such as excitotoxicity [13,14]. Excess activation of glutamate receptors results in deleterious consequences such as calcium buffering impairment, free radical generation, the mitochondrial permeability transition activation, and secondary excitotoxicity [15,16]. Glutamate excitotoxicity is a final common pathway leading to neuronal death and observed in many neurological disorders including stroke/ischemia, temporal lobe epilepsy, Parkinson’s disease, amyotrophic lateral sclerosis, and Alzheimer’s disease [17,18,19].

Stress also elevates the HPA axis activity and increases glucocorticoid secretion, which buffers stress (Figure 1). The hippocampus regulates the HPA axis activity and is involved in the negative feedback mechanism of glucocorticoid secretion. The regulation is linked to cognitive and emotional behavior [20]. The HPA axis activity is also elevated in normal aging [21,22] and neurological disorders [23]. Hippocampal neurons are vulnerable to the pathological elevation of the HPA axis activity, which is linked to the occurrence and progression of cognitive disorders [24]. Furthermore, there are correlations between increase in glucocorticoid secretion and dementia severity or hippocampal atrophy in patients with probable Alzheimer’s disease [25]. Correlations are also reported between increase in glucocorticoid secretion and depression severity or hippocampal atrophy in patients with depression [26].

The hippocampus is vulnerable to zinc deficiency [27] and neuropsychological symptoms associated with enhanced HPA axis activity are observed in zinc-deficient animals [28,29]. The evidence suggests that zinc-deficient animals are models for psychiatric disorders. To understand the validity of applying zinc-deficient animals as a behavioral abnormality model, this paper deals with the effect of antidepressive drugs and herbal medicines on hippocampal dysfunctions and behavioral abnormality, which are induced by enhanced HPA axis activity under dietary zinc deficiency.

Dietary zinc deficiency and stress decrease serum zinc level [3] and enhanced HPA axis activity [5,6,7]. The chronic increase in glucocorticoid secretion by enhanced HPA axis activity induces hippocampal dysfunction and behavioral abnormality, which may be models for depressive symptoms and behavioral and psychological symptoms of dementia (BPSD), and is linked to responsibility to glutamate excitotoxicity in the hippocampus (Figure 1).

## 2. Hippocampus as a Major Target of Glucocorticoids

The hippocampus is a major target of glucocorticoids and is enriched with corticosteroid receptors [30,31]. Mineralocorticoid receptors are usually occupied with low levels of cortisol in humans (corticosterone in rats). Glucocorticoid receptors are markedly activated after exposure to stress [32]. Glucocorticoids readily increase glutamate release from neuron terminals in the hippocampus via the mechanism that may involve membrane-associated mineralocorticoid receptors. As an indirect mechanism, glucocorticoids can modify glutamatergic neuron activity via cross talk with the endocannabinoid system [33]. The rapid effects of glucocorticoids on synaptic activity may be linked to the diverse effects on memory processes through synaptic plasticity in the hippocampus. Acute stress induces synaptic insertion of calcium-permeable α-amino-3-hydroxy-5-methyl-4-isoxazolepropionate (AMPA) receptors, which facilitates long-term potentiation (LTP) in the hippocampus [34]. On the other hand, corticosterone-mediated blockade of glutamate transporters leads to glutamate accumulation in the extracellular compartment at high levels when abnormal corticosterone secretion is induced under severe stress. Abnormal corticosterone secretion also induces excess release of glutamate from neuron terminals in the hippocampus [35,36]. The spillover of glutamate in the extracellular compartment impairs spatial memory retrieval.

Glucocorticoid concentration in the plasma is higher in aged animals than young animals [21]. The nocturnal levels of cortisol are increased in aged humans [22]. Furthermore, high cortisol levels are observed in Alzheimer’s disease and depression. In patients with Alzheimer’s disease, core symptoms, e.g., cognitive deficits and BPSD, e.g., aggression, hallucinations, disturbed behavior, and agitation are linked to dysregulation of the HPA axis activity [37,38]. Excess cortisol secretion is linked to cognitive decline in normal aging, in addition to neurological disorders such as dementia. Therefore, zinc deficiency may become a risk factor for the pathogenesis of Alzheimer’s disease and depression. Zinc-deficient animals may be models to assess the effect of drugs on hippocampal dysfunctions and behavioral abnormality, which are associated with elevated HPA axis activity.

## 3. Vulnerability of Hippocampus to Glutamate Excitotoxicity and Its Enhancement by Zinc Deficiency

It is well known that the hippocampus is vulnerable to glutamate excitotoxicity. Hippocampal neuronal death is reported in temporal lobe epilepsy, in which epileptic seizures frequently occur in the hippocampus [39]. When extracellular glutamate is increased in the hippocampus, the increase may trigger epileptic seizures in patients with complex partial epilepsy [40]. Brain zinc homeostasis is closely linked to the pathophysiology of epileptic seizures [41]. In global ischemia, potassium concentration transiently reaches 75 mM in the extracellular compartment and the increase leads to glutamate accumulation in the extracellular compartment [42]. Reversed operation of glial glutamate transporter, GLT-1, which may be induced with energy (ATP) depletion, is involved in the accumulation of extracellular glutamate in ischemia, which is crucial to excitotoxic death of neurons [43]. High K^+^-mediated increase in extracellular glutamate is enhanced in the hippocampus of zinc-deficient rats [10,44]. Neurological disorders, such as epilepsy and ischemia, seem to be aggravated by the enhanced glutamate excitotoxicity under zinc deficiency [11,45,46].

**Figure 2:**
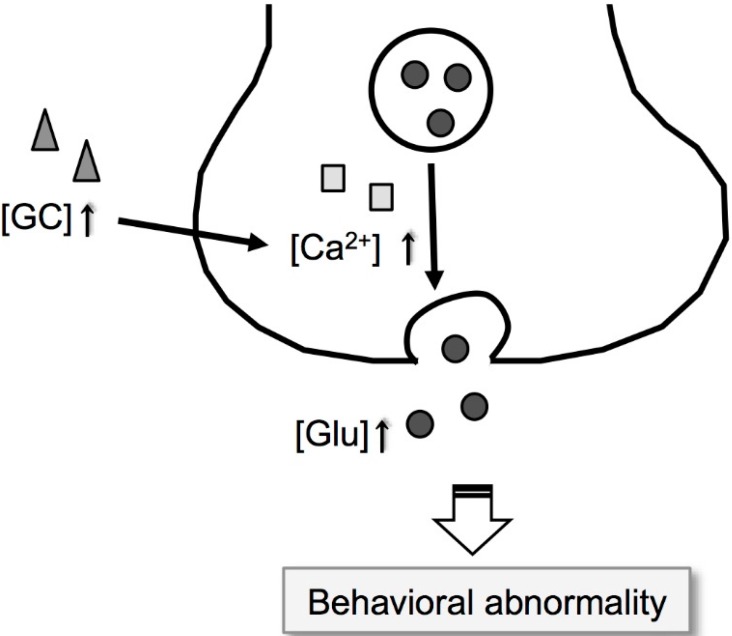
Modification of glutamate signaling via glucocorticoid signaling under dietary zinc deficiency. Extracellular glucocorticoid [GC] concentration in the hippocampus increased by dietary zinc deficiency can elevate glutamatergic neuron activity via modification of intracellular Ca^2+^ signaling as shown by the two long black arrows. Grey triangle, grey square, and grey circle show increases in extracellular GC, intracellular Ca^2+^, and extracellular glutamate, respectively.

Excess glucocorticoid secretion may modify glutamate neurotransmission in the hippocampus under zinc deficiency [47]. Glucocorticoids elevate voltage-dependent calcium conductance, in addition to calcium-dependent afterhyperpolarization [48,49]. Ca^2+^ mobilization is modified in hippocampal neurons with glucocorticoids; Ca^2+^ mobilization into the cytosolic compartment is increased by glucocorticoids, while its removal is decreased by glucocorticoids [50]. Cytosolic Ca^2+^ concentration in the hippocampus is increased in brain slices prepared from zinc-deficient animals [8,51]. Glucocorticoid-mediated modification of intracellular Ca^2+^ dynamics seems to be linked to hippocampal function under zinc deficiency (Figure 2). Presynaptic activity (exocytosis) is elevated at hippocampal mossy fiber synapses in brain slices prepared from zinc-deficient rats [44].

Modification of Ca^2+^ signaling via the increase in the basal level of Ca^2+^ is reported in hippocampal neurons of aged animals [52,53]. Serum zinc level is significantly lower in aged animals than in young animals, while zinc concentration in the brain of aged animals and humans is almost the same as that of young animals and humans [54,55,56,57]. Glucocorticoid secretion, which is increased by aging and zinc deficiency, is linked to glutamatergic neuron activity in the hippocampus. Thus, the increased secretion may contribute to susceptibility to glutamate excitotoxicity in elderly and zinc-deficient people (Figure 1).

## 4. Zinc Deficiency and Depression

The regulation of the HPA axis activity is affected in approximately 50% of human depressives [58]. Interestingly, it is reported that human depressives are zinc-deficient [59,60]. The increase in depression-like behavior has been reported in zinc-deficient mice [61] and rats [62]. The symptoms are observed without appreciably decreasing zinc concentration in the brain [63,64]. Depressive symptoms are also reported in sheep and goats [65]. Anxiety- and depression-like behavior is increased in mice and rats after repeated injections of corticosterone [66,67]. The contentious increase in corticosterone under dietary zinc deficiency may be linked to depression-like behavior.

Stress reduces hippocampal neurogenesis and reduced neurogenesis is associated with the pathophysiology of depression [68,69]. Antiglucocorticoids such as conventional antidepressants can ameliorate depressive symptoms via elevating hippocampal neurogenesis [70,71]. Decreased brain zinc availability under chronic zinc deficiency reduces hippocampal neurogenesis in mice and rats [72], and might be related to the pathophysiology of depression. Abnormal secretion of glucocorticoid induced by chronic stress has been observed in many neuropsychiatric disorders such as depression [73,74]. Neuropsychiatric disorders are exacerbated by exposure to stress. The hippocampus is vulnerable to stress-related disorders [30,31]. The interplay between glutamatergic neurotransmission in the hippocampus and the chronic or excess exposure to glucocorticoids plays a key role for pathogenesis of depression (Figure 1) [75].

Serum zinc concentration is decreased in patients with depression [59,76], while effective antidepressant treatment normalizes the decrease in serum zinc [77]. Zinc supplementation is effective for antidepressant therapy using drugs, such as selective serotonin reuptake inhibitors [78,79], while zinc deficiency reduces responsiveness to antidepressant drugs in mice [80]. Administration of glutamate receptor antagonists, including zinc, shows antidepressant effect in preclinical and clinical studies [81]. Zinc also has a robust effect on reversing behavioral alteration induced by chronic unpredictable stress in mice, possibly through the modulation of glutamatergic neurotransmission [82]. The decrease in serum zinc may be a state marker of depression [78].

## 5. Zinc Deficiency and Behavioral and Psychological Symptoms of Dementia (BPSD)

Externalizing behaviors, such as aggression, hyperactivity, and conduct disorder, have been viewed as a public health problem in childhood based on its etiology and outcome. Poor nutrition such as zinc deficiency is involved in the development of behavioral abnormality in childhood [83]. Zinc deficiency might be associated with assaultive behavior of young men. Plasma copper/zinc ratios are elevated in assaultive young males [84]. In experimental animals, social isolation is an effective method of inducing aggressive behavior. Social isolation-induced aggressive behavior is significantly increased in zinc-deficient young mice [9]. In contrast, neuropsychological behaviors are improved in school-age children with zinc supplementation [85,86].

Yokukansan is a traditional Japanese herbal medicine and a remedy for insomnia, irritability, and neurosis in children. The clinical effectiveness of Yokukansan has also been reported in patients with dementia including Alzheimer’s disease [87,88,89]. BPSD is a major problem for caregivers [90] and its severity is positively correlated with the care burden. Curing BPSD is important for both patients and caregivers [91,92]. More than 60% of patients with Alzheimer’s disease show agitation and aggression [93], which are the primary cause of hospitalization [94]. Yokukansan is an effective drug to cure BPSD [87,88,89,90,91]. Disturbed glutamatergic neuron activity in the hippocampus might underlie both BPSD and core symptoms [95]. On the basis of the evidence that zinc deficiency-induced modification of glucocorticoid secretion disturbs glutamatergic neuron activity in the hippocampus and induces behavioral abnormality (Figure 1) [96], zinc-deficient animals have been used to estimate the action mechanism of Yokukansan on BPSD. Administration of Yokukansan to zinc-deficient mice ameliorates social isolation-induced aggressive behavior, potentially via ameliorating abnormal glutamatergic neuron activity in the hippocampus [44,97,98,99]. Administration of Yokukansankachimpihange, a potential herbal medicine for BPSD, to zinc-deficient mice also ameliorates social isolation-induced aggressive behavior. The two common ingredients ameliorate abnormal glutamatergic neuron activity in the hippocampus [100]. It is likely that zinc-deficient animals are models to assess the effect of drugs on behavioral abnormality, such as BPSD.

## 6. Conclusions and Perspective

The evidence suggests that zinc-deficient animals are useful models for behavioral abnormality such as depressive symptoms and BPSD. The disturbance of glutamatergic neurotransmission in the hippocampus, which is induced by enhanced HPA axis activity under dietary zinc deficiency, underlies behavioral abnormality (Figure 1 and Figure 2).

On the other hand, diabetes mellitus chronically increase the HPA axis activity, which may contribute to insulin resistance [101]. Depression-like behavior is observed in GPR39 (Zn^2+^-sensing receptor) knockout mice without an increase in serum corticosterone [102]. Huang et al. [103] report that the serious effects on rat brain development induced by marginal zinc deficiency may not be linked with fetal exposure to excess maternal glucocorticoids. Therefore, further investigation on the involvement of other endocrine systems in behavioral abnormality caused under zinc deficiency is necessary for validity as experimental models.
